# Paternal deprivation affects the functional maturation of corticotropin-releasing hormone (CRH)- and calbindin-D28k-expressing neurons in the bed nucleus of the stria terminalis (BNST) of the biparental *Octodon degus*

**DOI:** 10.1007/s00429-013-0617-4

**Published:** 2013-08-03

**Authors:** Tomasz Gos, Jay Schulkin, Anna Gos, Joerg Bock, Gerd Poeggel, Katharina Braun

**Affiliations:** 1 Institute of Forensic Medicine, Medical University of Gdansk, ul. Sklodowskiej-Curie 3a, 80-210 Gdansk, Poland; 2 Department of Neuroscience, Center for the Brain Basis of Cognition, Georgetown University, Washington DC, 20007 USA; 3 Department of Obstetrics and Gynecology, University of Washington, Seattle, WA 98195 USA; 4 Institute for Biology, Human Biology, University of Leipzig, Talstraße 33, 04103 Leipzig, Germany; 5 Department of Zoology/Developmental Neurobiology, Institute of Biology, Otto von Guericke University, Leipziger Straße 44, 39120 Magdeburg, Germany; 6 Project Group Epigenetics and Structural Plasticity, Institute of Biology, Otto von Guericke University, 39120 Magdeburg, Germany; 7 Center for Behavioral Brain Sciences (CBBS), Magdeburg, Germany

**Keywords:** Parental behavior, Development, Corticotropin-releasing factor, Quantitative immunocytochemistry

## Abstract

While the critical role of maternal care on the development of brain and behavior of the offspring has been extensively studied, our knowledge about the importance of paternal care for brain development of his offspring is still comparatively scarce. The aim of this study in the biparental caviomorph rodent *Octodon degus* was to analyze the impact of paternal care on the development of corticotropin-releasing hormone (CRH)-expressing neurons in the bed nucleus of the stria terminalis (BNST) and hypothalamic paraventricular nucleus (PVN). Both brain areas are key players in neuronal circuits that regulate hypothalamic–pituitary–adrenal axis (HPA) activity. At the age of postnatal day (PND) 21, we found that paternal deprivation resulted in a decreased density of CRH-containing neurons in the medial, but not in the lateral BNST, whereas no changes were observed in the PVN. These deprivation-induced changes were still prominent in adulthood. At PND 21, the density of Ca-binding protein calbindin D28K (CaBP-D28K)-expressing neurons was specifically increased in the medial, but not lateral BNST of father-deprived animals. In contrast, adult father-deprived animals show significantly decreased density of CaBP-D28K-expressing neurons in the lateral, but not medial BNST. Taken together, these results may have important implications for our understanding of the experience-driven development of neural circuits that regulate HPA activity mediating acute responses to stress and chronic anxiety.

## Introduction

The activation of the hypothalamic–pituitary–adrenal (HPA) axis represents an essential mechanism for maintaining homeostasis in response to stress exposure. Specific neuron populations in the hypothalamic paraventricular nucleus (PVN) synthesize corticotropin-releasing hormone (CRH) that is released into the hypophysial portal system and triggers adrenocorticotropin (ACTH) secretion from the anterior pituitary, which in turn activates the secretion of corticosterone from the adrenals into the blood stream (Herman et al. 2005). Limbic and prefrontal cortical regions including the amygdala, hippocampus and medial prefrontal cortex have substantial influence on the HPA axis (see review by Herman et al. 2005), and dysfunction of these regions is implicated in the etiology of stress-related disorders. Since many stress-related mood disorders originate from emotional experience during childhood, it is critical to identify the environmental factors, which affect the development of those neuronal circuits, modulating HPA sensitivity.

Studies on humans and experimental investigations on animal models revealed that emotional deprivation or stressful family settings were a major source of vulnerability to develop behavioral and mental disorders in later life. Most studies have focused on the critical impact of maternal care (or the lack of) on behavioral and brain development. This is somewhat surprising in view of animal species, including humans, where it has been shown that the presence of the father in the family and his investment in paternal care are essential for the development of his offspring (Atzil et al. 2012; Blanz et al. 1986; Dewsbury 1985; Sherman and Neff 2003; Vieira and Brown 2003; Wynne-Edwards and Lisk 1989; Schradin and Pillay 2003). An ideal animal model to systematically and quantitatively study the impact of paternal care on the neuronal and behavioral development of the offspring is the trumpet-tailed rat *Octodon degus*, a biparental South American rodent (Fuchs et al. 2010; Pinkernelle et al. 2009; Helmeke et al. 2009; Ebensperger et al. 2010). Studies on humans and animal models revealed that paternal care significantly affected the development of cortico-limbic synaptic circuits (Helmeke et al. 2009; Braun et al. 2011; Seidel et al. 2011; Narita et al. 2010). In *O. degus*, we observed that paternal care interfered with the developmental expression patterns of CRH- and Ca-binding protein calbindin D28K (CaBP-D28K)-expressing neurons in prefrontal and limbic brain regions of the offspring (Braun et al. 2011; Seidel et al. 2011).

Corticotropin-releasing hormone is the major neurohormone regulating the vertebrate endocrine response to diverse events, including adversity. In addition to the hypothalamus, CRH is expressed in limbic structures, prefrontal, and cingulate cortex and brainstem nuclei associated with autonomic functions. Other regions with predominant CRH-containing neurons are the lateral bed nucleus of the stria terminalis (BNST) and regions of the amygdala (Swanson et al. 1983). Less is known about the exact function of the neuron-specific CaBP-D28K, which may act as an intracellular calcium buffer and thereby mediate the differential vulnerability of specific neuron subpopulations exposed to excitotoxic conditions such as stroke and seizures, and in neurodegenerative diseases. A host of publications have revealed the great value of this Ca-binding protein as a neuronal marker for identifying distinct neuronal subpopulations (reviews by Braun et al. 1991; Heizmann and Braun 1992; Braun 1990; Celio 1990).

The semi-deprived environment in fatherless families allowed us to test the hypothesis that paternal care essentially affects the development of specific neuronal subpopulations and to identify affected brain regions. In humans and other animals, parents provide the earliest socio-emotional environment. Recent studies show that socio-emotional stimulation, in particular during the interaction with the mother, is essential for the development of endocrine functions, brain wiring and behavior; however, much less is known about the contribution of paternal care. What are the underlying mechanisms that mediate the neuronal changes resulting from paternal deprivation? The importance of the sensory environment and physical exercise during ‘‘experience-expectant’’ neuronal development has been demonstrated for the functional maturation of sensory and motor systems in a variety of model systems; however, less is known about the importance of socio-emotional stimulation on the functional development of emotional brain circuits. In this study, we tested the hypothesis that paternal deprivation affects the development of CRH- and CaBP-D28k-expressing neurons, the BNST and the PVN as hypothalamic key players of HPA stress modulation.

## Materials and methods

### Animals

The degus used in this study were bred in our colony (Leibniz Institute for Neurobiology, Magdeburg). The families consisted of an adult couple and their offspring and were housed in wire cages (5,100 × 4,200 × 6,800 mm) in air-conditioned rooms with an average temperature of 22 °C, and an artificial light/dark cycle (12/12 h). Fresh drinking water, rat diet pellets (SSNIFF special diets GmbH, Soest, Germany) and vegetables were available ad libitum. All experiments were performed in accordance with the European Community’s Council Directive of 24th November 1986 (86/609/EEC), and according to the German guidelines for the care and use of animals in laboratory research the experimental protocols were approved by an ethics committee.

### Experimental groups

Twelve juvenile (postnatal day, PND 21) and 12 adult (PND 90) male degus were used for the quantitative histological analysis. For each manipulation, the entire litter was treated identically. The following rearing conditions were analyzed:

(i) *Biparental families*:

(+F/21, *N* = 6) The pups were raised together with both parents and their siblings until PND 21.

(+F/90, *N* = 6) The pups were raised together with both parents and their siblings until PND 45 (time of puberty). For reasons of potential sexual maturity of the siblings, two male siblings of each litter were separated from their parents at PND 46 and housed together until PND 90.

(ii) *Father-deprived animals (single-mother families):* the father was removed from the nest when the pups were 1 day old.

(−F/21, *N* = 6) The pups were raised together with the dam and siblings until PND 21.

(−F/90, *N* = 6) The pups were raised together with the dam and siblings until PND 45 (time of puberty). For reasons of potential sexual maturity of the siblings, two male siblings of each litter were separated from their parents at PND 46 and housed together until PND 90.

### Quantitative immunohistochemistry

At the age of 22 (juvenile group) and 91 days (adult group), the pups were removed from their home cage and anesthetized with Ketanest/Rompun (1:4, 7 ml/kg, intraperitonally) (Ketanest: Porke-Davis, Berlin, Germany; Rompun: Bayer, Leverkusen, Germany) and subsequently transcardially perfused with 50 ml Tyrode’s buffer and 1 % Liquemin (Roche, Grenzach-Wyhlen, Germany), followed by 150 ml 4 % paraformaldehyde in 0.1 M sodium acetate buffer (pH = 6.5) and 300–400 ml 4 % paraformaldehyde in 0.1 M sodium borate buffer (pH = 9.3). Thereafter, the brains were removed from the skull, weighed and postfixed at 4 °C for 1 h in the last fixative. In the next step, the brains were transferred into 0.1 M phosphate-buffered saline (PBS, pH = 7.4) and cut into alternating frontal sections using a Vibratome (Leica Instruments GmbH, Nußloch, Germany) and collected in PBS. For quenching endogenous peroxidase activity, the brain sections were treated with 10 % methanol and 1 % hydrogen peroxide for 45 min and after several washing steps preincubated in PBS containing 2 % normal goat serum (DAKO, Hamburg, Germany) and 0.3 % Triton-X-100 (Sigma-Aldrich, Deisenhofen, Germany) for 1 h. Alternate sections were incubated with either primary antibody of (1) rabbit-anti-CRH (1:8,000; Peninsula Laboratories, Inc., USA) or (2) mouse-anti-CaBP-D28k (1:5,000; SWANT, Switzerland) diluted in PBS containing 1 % normal goat serum, 0.05 % Triton-X-100 at 4 °C for 48 h. In control sections, the primary antibody was omitted. After washing in PBS, the sections were incubated with biotinylated secondary goat-anti-rabbit antibody (1:200) for 2 h, followed by incubation with streptavidin–peroxidase complex (Sigma-Aldrich, Deisenhofen, Germany) at 1:200 in phosphate buffer (PB, pH = 7.6) for 2 h. After subsequent washes in PB and Tris–HCl buffer (pH = 7.6) (Sigma-Aldrich, Deisenhofen, Germany), the sections were reacted in a DAB solution of 0.05 % 3,3’-diaminobenzidine hydrochloride, 2 % ammonium nickel sulfate (Riedel-de Haen AG, Seelze, Germany) and 1 % hydrogen peroxide in Tris–HCl buffer. The stained slices were mounted on chrome alum-coated slides, air dried, dehydrated in graded series of alcohol (70 %, 96 %, isopropanol, xylene) and coverslipped with Histoclear in Histomount (Life Sciences International, Frankfurt, Germany).

The control sections displayed no specific staining. For quantitative light microscopy the degu brain atlas (Wright and Kern 1992) and the rat brain atlas (Paxinos and Watson 2004) were used for the histological identification of the BNST and its subregions and for the localization of the PVN.

For each animal and immunocytochemical marker, three to five sections at a distance of 200 μm and within the regions of interest defined frames (in μm^2^) were selected and scanned at a magnification of 20× objective using a computer-connected Zeiss Imager.Z1 microscope equipped with a digital AxioCam MRm camera. Quantitative analysis was conducted using the AxioVision software.

### Statistical analysis

A three-way-ANOVA with two between-subject factors (rearing condition and age) and one within-subject factor (hemisphere) was used to assess possible hemispheric effects as well as interactions between the hemisphere and the between-subject factors (SPSS version 15.0, SPSS Inc.). Since no hemispheric effects and interactions were detectable, the hemispheric data were pooled and a two-way ANOVA (GraphPad Prism version 5.00, GraphPad Software, San Diego, California, USA) was applied with rearing conditions and age as factors. Bonferroni post hoc test was applied to compare between groups. For all comparisons, the level of significance was set to *p* ≤ 0.05. All data are presented as mean ± SEM.

### Preparation of figures

Digital images were created by using Adobe Photoshop 7.0 (Adobe Systems Incorporated, USA) and assembled into montages. General adjustments of color, contrast and brightness were made.

## Results

### Bed nucleus of the stria terminalis (BNST)

#### Corticotropin-releasing factor

**Fig. 1:**
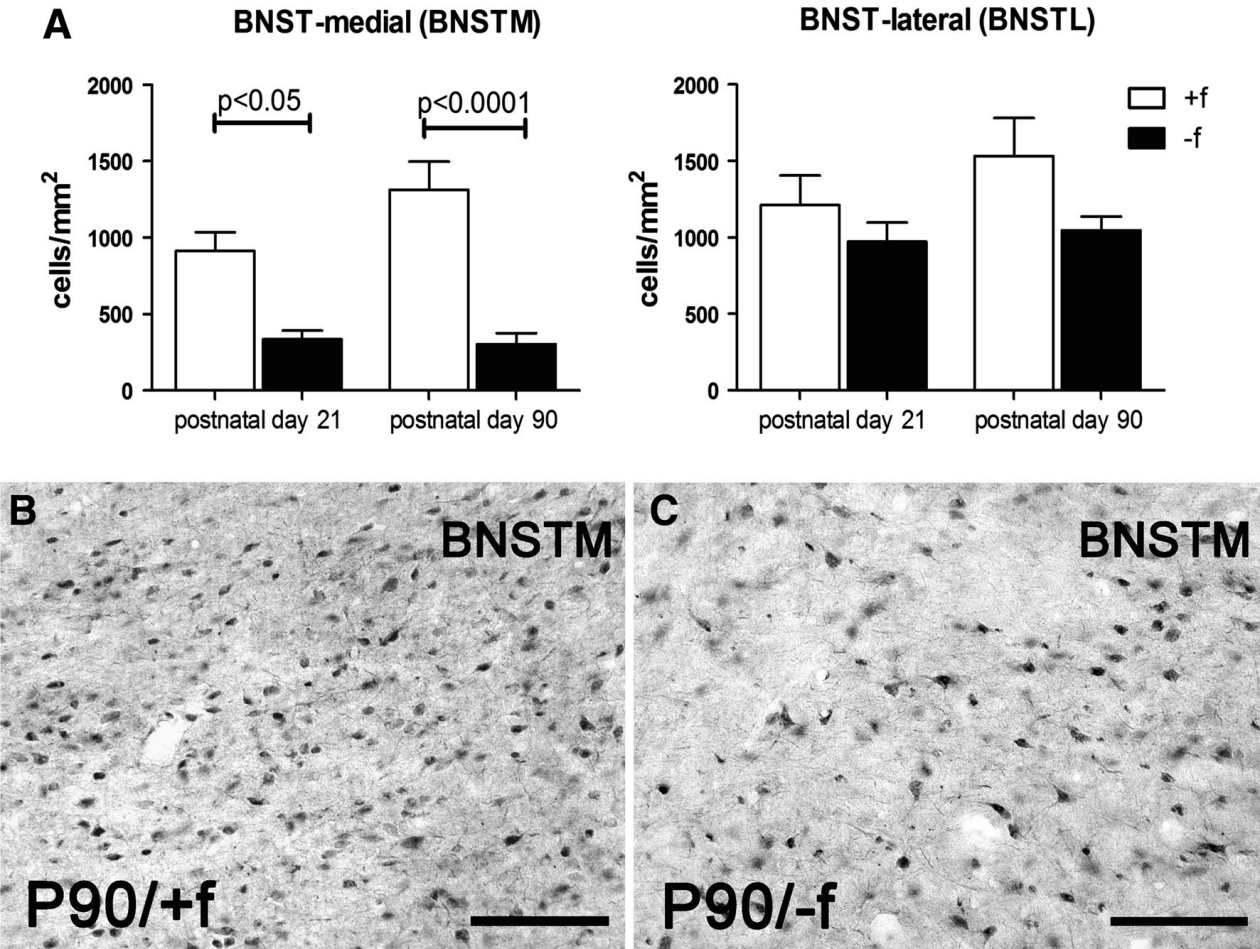
Changes in CRH-expressing neuron density in the BNST in response to paternal deprivation. **a** Differential changes in the medial (*left*) and lateral (*right*) BNST. **b**, **c** Photomicrograph showing the reduction of CRH-positive neurons in the medial BNST (BNSTM) of adult father-deprived (*−f*) compared to biparentally reared (*+f*) offspring. *Bar* in **b**, **c** = 100 μm

Two-way ANOVA revealed a significant effect of rearing condition (*F*_1,18_ = 37.55, *p* < 0.0001); no significant effect was found for age (*F*_1,18_ = 2.033) and no interaction of age × rearing condition (*F*_1,18_ = 2.791) was detected for the medial BNST. Posthoc test revealed that in both age groups, the father-deprived juvenile (*p* < 0.05) and adult (*p* < 0.001) animals displayed a significantly lower density of CRF-expressing neurons in the medial BNST compared to biparentally raised animals (Fig. 1). In contrast, no significant difference was observed in the lateral BNST (Fig. 1).

#### Calbindin-D28K

**Fig. 2:**
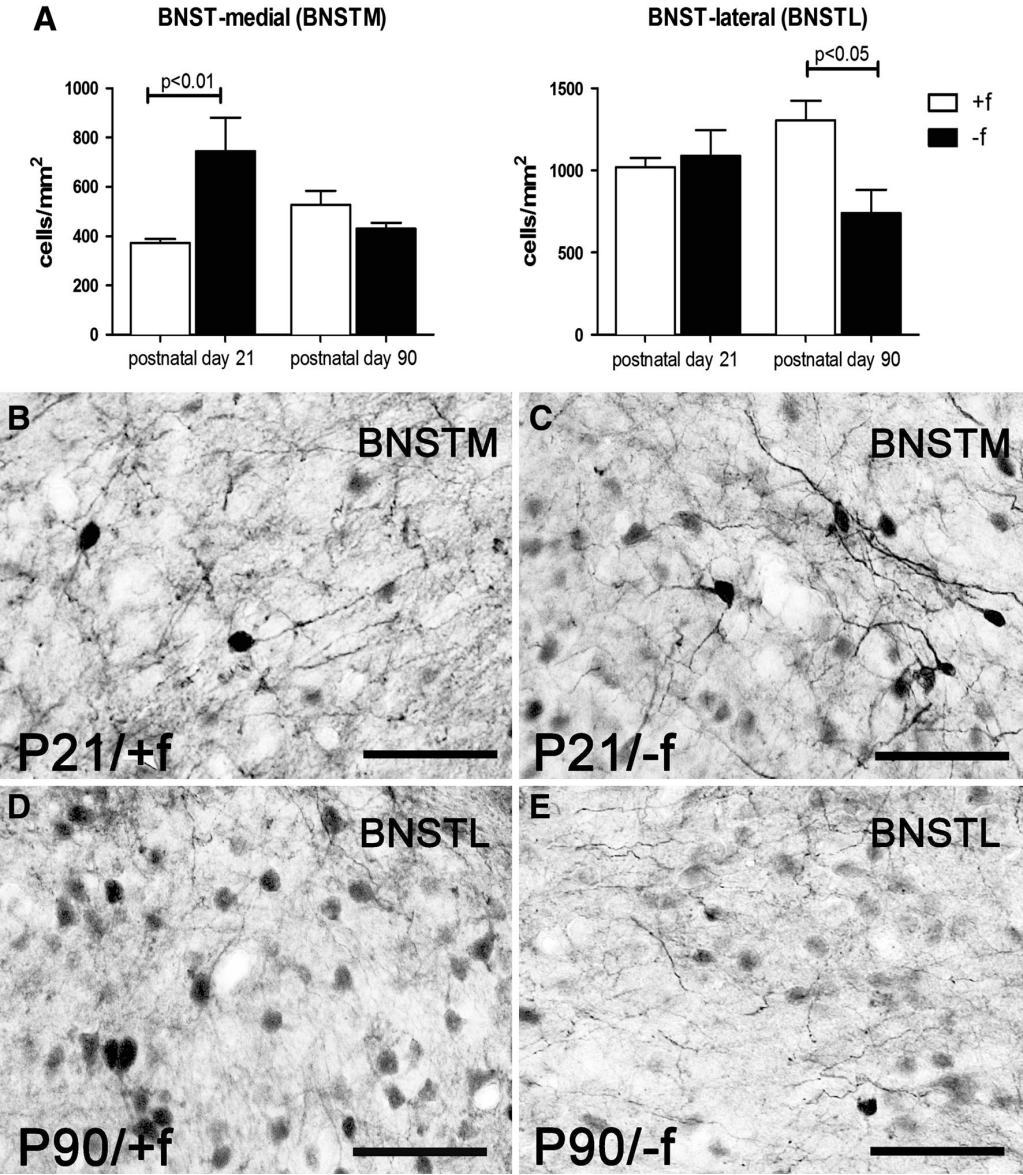
Changes in CaBP-D28K-expressing neuron density in the BNST in response to paternal deprivation. **a** Differential changes in the medial (*left*) and lateral (*right*) BNST. **b**, **c** Photomicrograph showing the increase of CaBP-D28K-expressing neurons in the medial BNST (BNSTM) of juvenile father-deprived (*−f*) compared to biparentally reared (*+f*) offspring. **d**, **e** Photomicrograph showing the reduction of CaBP-D28K-expressing neurons in the lateral BNST (BNSTL) of adult father-deprived (*−f*) compared to biparentally reared (*+f*) offspring. *Bar* in **b**–**e** = 50 μm

Two-way ANOVA revealed in the medial BNST an interaction of age × rearing condition (*F*_1,18_ = 8.115, *p* = 0.01); no significant effect was found for factors age (*F*_1,18_ = 2.794) and rearing condition (*F*_1,18_ = 0.945). For the P21 age group, a significant increase (*p* < 0.05) in the density of CaBP-D28K-immunoreactive neurons was found in the father-deprived group compared to the biparentally raised group (Fig. 2).

In the lateral BNST, an interaction of age × rearing condition was observed (*F*_1,18_ = 6.016, *p* = 0.025); no significant effects of age (*F*_1,18_ = 0.055) and rearing condition (*F*_1,18_ = 3.711) were found. For the P90 age group, a significant decrease (*p* < 0.05) in the density of CaBP-D28K-immunoreactive neurons was found in the father-deprived group compared to the biparentally raised group (Fig. 2).

### Nucleus paraventricularis (PVN)

No significant differences were found between the rearing and age groups for the density of CRH- or CaBP-D28K-expressing neurons in the PVN.

## Discussion

We show here for the first time in the biparental caviomorph rodent *O. degus* that paternal care strongly affects the development of CRH- and CaBP-D28K-expressing neurons in hypothalamic regions of the offspring in a region- and age-specific manner. Already at the age of 3 weeks, the density of CRH-containing neurons was decreased in the medial, but not in the lateral BNST of paternally deprived offspring. These changes were still prominent in adulthood. The density of CaBP-D28k-expressing neurons was specifically increased in the medial, but not lateral BNST of 3-week-old father-deprived animals. In contrast, adult father-deprived animals display significantly decreased density of CaBP-D28K-expressing neurons in the lateral, but not medial BNST. In the PVN, the expression of these neuronal subpopulations was not affected by paternal deprivation.

The medial and lateral compartments of the BNST, which are parts of extended amygdala involved in the regulation of autonomic and neuroendocrine systems (Alheid 2003), display differences between their functional properties. First, the anteromedial group heavily and directly innervates neuroendocrine components of the hypothalamus, whereas the anterolateral group does not. Second, the anteromedial group much more clearly innervates brainstem regions associated with pelvic nerve functions. Third, the anterolateral group innervates much more strikingly hindbrain sensory and motor regions associated with the vagus nerve. Fourth, overall, the anterolateral group tends to innervate regions associated with energy balance homeostasis and feeding behavior, whereas the anteromedial group tends to innervate regions (including the subfornical organ and median preoptic nucleus) associated with water balance homeostasis and drinking behavior as well as with energy balance homeostasis and feeding behavior (Dong and Swanson 2006). According to neurophysiological studies on stress axis, the lateral BNST stimulation induces the activation of the PVN, whereas the medial BNST exerts the direct GABAergic inhibitory input on this structure (for a review see Hammack et al. 2010).

**Fig. 3:**
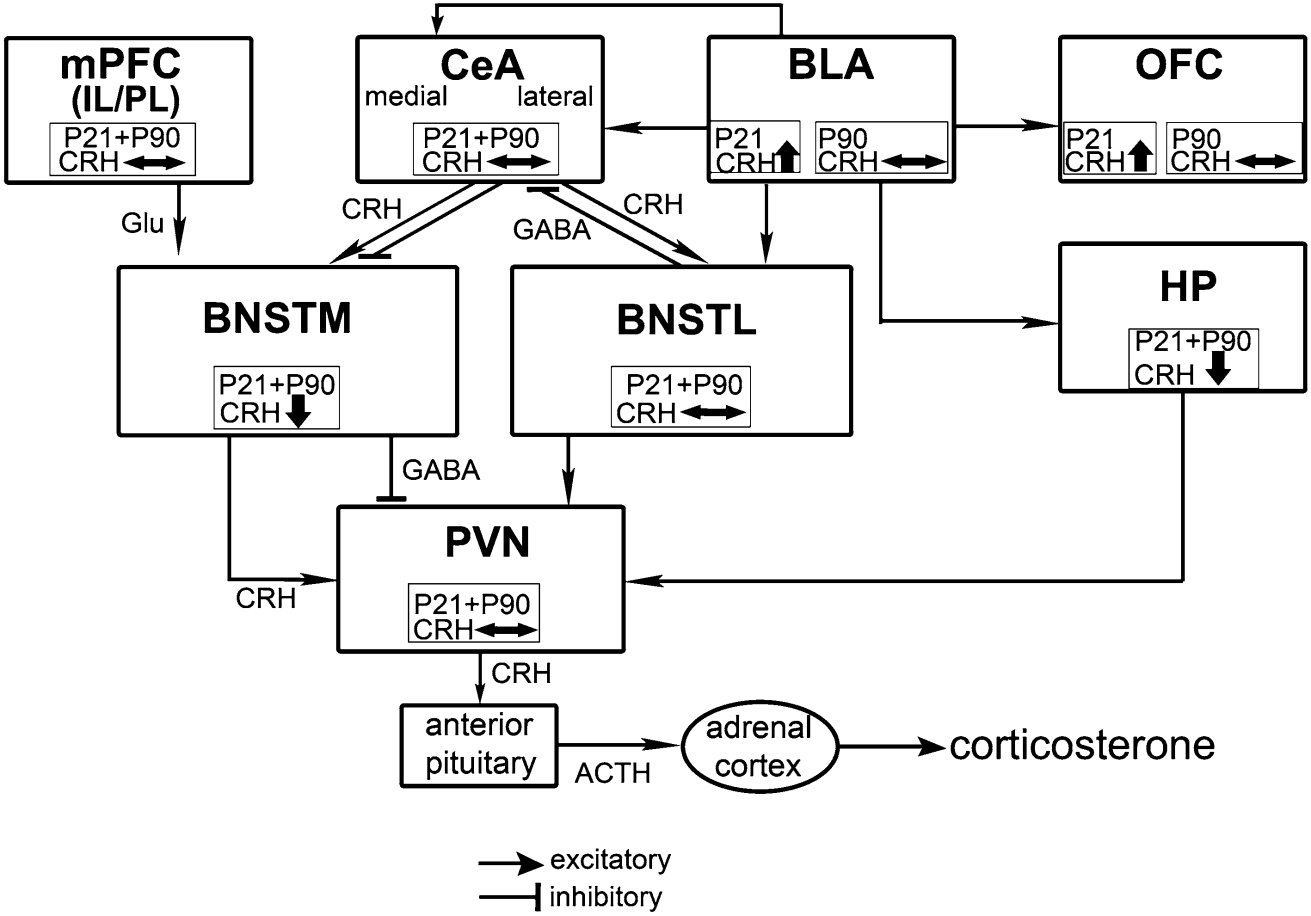
Simplified diagram of prefronto-limbic–HPA pathway, summarizing the age- and region-specific changes in CRH-neurons induced by paternal deprivation. Not all known connections between the different brain nuclei are shown. Assuming that, similar to findings in cortical regions, the CaBP-D28K-immunoreactive neurons represent GABAergic projecting neurons, we speculate that this neuronal subpopulation in the medial BNST conveys a regulatory input from limbic structures on the PVN (Dent et al. 2007). The increase of this presumably inhibitory GABAergic transmission located in the medial BNST suggested by our results and findings in other stress and deprivation models (Dent et al. 2007; Bowers et al. 1998) may indicate an attenuation of inhibitory projection from the medial BNST resulting in a disinhibition of the PVN, which is transiently observed in the father-deprived juvenile offspring. Due to reciprocal projections providing positive feedback between structures of extended amygdala and the PVN (Champagne et al. 1998), the presumed up-regulation of the PVN in our model (Fig. 3) may in turn lead to hyperactivity of the lateral BNST suggested by the decreased density of CaBP-D28K-immunopositive neurons observed in this compartment in adult animals. The net effect of hypothesized diathesis as a consequence of paternal deprivation should be a prolonged up-regulation of stress reactivity and anxiety related to the functional imbalance between BNST compartments, i.e., the hypoactivity of the medial and the hyperactivity of the lateral compartment

The critical role of CRH-mediated circuits (Fig. 3) has been demonstrated in diverse experiments, which have revealed that the activation of CRH circuits elicits behavioral responses that are typically observed during states of fear or anxiety (Rosen and Schulkin 1998; Davis and Shi 1999). One hypothesis is that CRH in the amygdala is tied more specifically to fear and CRH in the bed nucleus to anxiety (Schulkin et al. 2005; Walker et al. 2003). In other words, while the central amygdala has been shown to mediate fast fear responses toward specific sensory cues, the BNST may be predominantly involved in extended states of anxiety in response to diffuse environmental stimuli (Nagy and Pare 2008; Davis and Shi 1999). Whereas these two emotional states are closely linked, they also differ in important ways. Fear is a naturally occurring adaptive change evoked by potentially threatening stimuli (Ledoux 1995; Rosen and Schulkin 1998). While fear is elicited by an identifiable stimulus and subsides shortly after its offset, anxiety may not be clearly associated with a defined stimulus, may last for long periods once activated and lack adaptive significance (Lee and Davis 1997).

Our results are quite intriguing in view of these possibly coordinated functional interactions between the amygdala and the BNST as they indicate that CRH neurons in the medial region of the BNST are chronically altered by paternal deprivation, whereas these changes are not observed in the lateral BNST and in the central amygdala (see Fig. 3) (Seidel et al. 2011). These findings add to the literature on the separation of the two systems “fear” and “anxiety” in the brain: one system perhaps tied to specific fear function and the other to more anxious sensibility (Davis and Shi 1999). The integration of the HPA-mediated stress response involves interactions between stress-sensitive prefrontal and limbic brain regions, neuroendocrine hypothalamic neurons and their feedback regulation of HPA activity (Fig. 3).

Our findings suggest that the father-deprived animals display dysfunctions of the “anxiety”-related subcircuits. This view is supported by our previous findings, which demonstrate that, in addition to the effects on CRH-expressing neurons in the medial BNST, paternal deprivation induces region- and age-specific changes of CRH-expressing neurons in the prefrontal and limbic regions (Fig. 3). Furthermore, preliminary experiments using the open field test indicate that the father-deprived offspring displays elevated anxiety, indicated by a decrease in activity in the center of the open field arena (data not shown). In juvenile animals, the orbitofrontal cortex and the basolateral amygdala displayed an elevated density of CRH-positive neurons. These changes disappear until adulthood, indicating that social experience during later life may “compensate” for the juvenile deprivation. On the other hand, even transient changes occurring within short developmental time windows can still result in long-lasting effects, including the “hypofunction” of the medial BNST. In contrast, the CA1 region of the hippocampal formation and the medial BNST show lasting reduction of the density of CRH-expressing neurons in father-deprived animals, whereas the subregions of the medial prefrontal cortex remain unaltered (see Fig. 3) (Seidel et al. 2011).

Even though CaBP-D28K-immunoreactive neurons represent a remarkably large neuron population in the BNST, surprisingly little is known about their specific physiological function (Budefeld et al. 2008; Gilmore et al. 2012). The EF-hand Ca-binding protein CaBP-D28K has been used as immunocytochemical marker to identify specific subpopulations of inhibitory interneurons in the neocortex, the hippocampal formation and cerebellum (Heizmann and Braun 1992; Braun 1990; Schmidt 2012). A few studies provide evidence that the overexpression of CaBP-D28K may be neuroprotective (Yuan et al. 2013) and that this cytoplasmatic Ca-buffer is regulated in relation to neuronal activity (Baimbridge et al. 1985). Interestingly, the lack of paternal care also affects the development of CaBP-D28K-expressing neurons in a region- and age-dependent manner (Braun et al. 2011); however, the developmental patterns differ from those observed for the CRH-expressing neurons. Transient (i.e., only in juveniles) elevation of CaBP-D28K-expressing neurons was observed in the orbitofrontal cortex, hippocampal formation and the medial BNST. These changes were no longer seen in adulthood. Interestingly, those regions in which no changes were seen in the juveniles showed “delayed” changes in adulthood, where a reduction of CaBP-D28K-neuron density was found in the lateral BNST and in the central and basolateral amygdala (Braun et al. 2011). Although no permanent changes of CaBP-D28K-neuron density were seen in the subregions of the medial prefrontal cortex (Braun et al. 2011), it is tempting to speculate that—considering the anatomical connection between the amygdala and the PFC—the experience-induced changes in the amygdala (Braun et al. 2011) may also affect the functionality of the PFC.

### Methodological considerations

Deprivation-induced changes might reflect (1) a true change in the number/density of CRH- or CaBP-D28k-immunoreactive neurons (e.g., via apoptosis/neurogenesis, impaired migration) and/or (2) altered physiological activity levels of these neuron populations, which might be reflected by their expression of CaBPD28k or CRH, respectively. The expression of CaPB-D28k is activity dependent (e.g., (Iacopino and Christakos 1990; Lowenstein et al. 1991); thus, neurons in which the concentration of this Ca-binding protein falls below or rises above the immunocytochemical detection limit may histologically disappear or appear, respectively.
